# Internet-Based Cognitive Behavioral Therapy for Children and Adolescents With Dental or Injection Phobia: Randomized Controlled Trial

**DOI:** 10.2196/42322

**Published:** 2024-02-21

**Authors:** Robert Schibbye, Erik Hedman-Lagerlöf, Viktor Kaldo, Göran Dahllöf, Shervin Shahnavaz

**Affiliations:** 1 Department of Dental Medicine Karolinska Institutet Huddinge Sweden; 2 Center of Pediatric Oral Health Stockholm Sweden; 3 Department of Clinical Neuroscience Karolinska Institutet Stockholm Sweden; 4 Centre for Psychiatry Research Department of Clinical Neuroscience Karolinska Institutet, & Stockholm Health Care Services Region Stockholm Sweden; 5 Department of Psychology Faculty of Health and Life Sciences Linnaeus University Växjö Sweden; 6 Center for Oral Health Services and Research Mid-Norway (TkMidt) Trondheim Norway

**Keywords:** adolescents, cognitive behavioral therapy, CBT, children, dental anxiety, dental fear, internet, specific phobia

## Abstract

**Background:**

Dental phobia (DP) and injection phobia (IP) are common in children and adolescents and are considered some of the biggest obstacles to successful treatment in pediatric dentistry. Cognitive behavioral therapy (CBT) is an evidence-based treatment for anxiety and phobias. As the availability of CBT in dentistry is low, internet-based CBT (ICBT) was developed. Open trials have shown that ICBT is a promising intervention, but randomized trials are lacking.

**Objective:**

This randomized controlled trial tests whether therapist-guided ICBT supported by a parent could reduce fear, allowing children and adolescents with DP or IP to receive dental treatment.

**Methods:**

We enrolled 33 participants (mean age 11.2, SD 1.9 y) whom a clinical psychologist had diagnosed with DP, IP, or both. After inclusion, participants were randomized to either ICBT (17/33, 52%) or a control group of children on a waitlist (16/33, 48%). ICBT was based on exposure therapy and comprised a 12-week at-home program combined with visits to their regular dental clinic. Participants corresponded weekly with their therapist after completing each module, and 1 parent was designated as a coach to support the child in the assignments during treatment. All participants completed measurements of the outcome variables before treatment start and after 12 weeks (at treatment completion). The measurements included a structured diagnostic interview with a clinical psychologist. Our primary outcome measure was the Picture-Guided Behavioral Avoidance Test (PG-BAT), which assesses the ability to approach 17 dental clinical procedures, and a positive clinical diagnosis. Secondary outcome measures included self-report questionnaires that measured self-efficacy and levels of dental and injection anxiety. The children and their parents completed the questionnaires.

**Results:**

All participants underwent the 12-week follow-up. After treatment, 41% (7/17) of the participants in the ICBT group no longer met the diagnostic criteria for DP or IP, whereas all participants in the control group did (*P*=.004). Repeated-measure ANOVAs showed that ICBT led to greater improvements on the PG-BAT compared with the control group; between-group effect sizes for the Cohen *d* were 1.6 (*P*<.001) for the child-rated PG-BAT and 1.0 (*P*=.009) for the parent-rated PG-BAT. Reductions in our secondary outcomes—dental fear and anxiety (*P*<.001), negative cognitions (*P*=.001), and injection fear (*P*=.011)—as well as improvements in self-efficacy (*P*<.001), were all significantly greater among children in the ICBT group than in the controls. No participants reported adverse events.

**Conclusions:**

ICBT seems to be an effective treatment for DP and IP in children and adolescents. It reduced fear and anxiety and enabled participants to willingly receive dental treatment. ICBT should be seriously considered in clinical practice to increase accessibility; this therapy may reduce the need for sedation and restraint and lead to better dental health in children and adolescents.

**Trial Registration:**

ClinicalTrials.gov NCT02588079; https://clinicaltrials.gov/study/NCT02588079

## Introduction

### Background

Dental fear and anxiety (DFA) refers to strong negative emotions associated with dental treatment [1]. In children and adolescents, the prevalence of DFA is 24%, making it a common clinical problem in pediatric dentistry [2]. Dental phobia (DP) and injection phobia (IP) are specific phobias classified as psychiatric disorders in *the Diagnostic and Statistical Manual of Mental Disorders* [3]. A specific phobia is defined as a marked and persistent fear that is excessive or unreasonable, in this case concerning the dentist, dental care in general, or a specific part of the dental treatment. The phobic object or situation is either avoided or endured with intense anxiety or distress. The fear is also persistent and typically lasts for at least 6 months, interfering significantly with the person’s normal functioning [3]. In dental care, if the phobia only pertains to receiving an injection but not to any other situation or object, it should be referred to as an IP if the fear of injections is general (ie, the fear is triggered by all types of injections), and it should be referred to as intraoral IP (a subtype of IP) if the fear only pertains to injections in the mouth. Both DP and IP are classified as subtypes of blood-injury-injection phobia, that is, a specific phobia related to seeing blood, injections, injuries, and disability or exposure to these or similar medical procedures [4]. It has been shown that 20% of children meet the diagnostic criteria for blood-injury-injection phobia at least once between the ages of 4 and 14 years [5]. DFA and DP or IP often lead to avoidance of dental care, which can cause decreased dental and general health [6].

Systematic reviews [1,7] have observed that traditional methods of treating pediatric patients with DFA, DP, or IP (ie, tell-show-do, restraint of some sort, sedation with midazolam or nitrous oxide, and general anesthesia) show little scientific evidence of treating the fear itself. These methods may be effective for preventing the development of DFA, DP, or IP before the first dental treatment or temporarily enabling dental treatment in groups of patients with anxiety and fear. However, they are ineffective in patients with severe DFA or previous negative treatment experiences, such as those with DP or IP. One reason may be that they do not incorporate exposure-based cognitive behavioral therapy (CBT), which is arguably the gold-standard method for treating specific phobias. Another reason is that they were not developed using a multidisciplinary approach that combines findings from the fields of both dental care and psychological science on reduction of anxiety and fear [1].

Stronger forms of DFA, DP, and IP are primarily learned through adverse events (ie, painful treatment procedures) in dental or general health care contexts, which results in fear being associated with dental care or specific objects or procedures, a process called respondent or classic conditioning [8,9]. CBT treatments are widely recognized as the most effective treatment for specific phobias and anxieties, but they are rarely implemented in dental practice [10]. The focus of CBT in the pediatric dental care setting is to reduce patients’ anxiety so that they can willingly receive treatment without the need for sedation or restraint. CBT, which is normally administered face-to-face by a trained psychotherapist, has been shown to be effective for both adults [11,12] and children and adolescents [13-16] with DFA, DP, and IP. However, accessibility to and use of CBT are generally low in dentistry. Common barriers include a lack of trained CBT therapists, high costs for the family if the child is treated in private psychotherapeutic services, and time constraints (ie, it might be difficult for parents to take time off from work for an extended period for weekly visits to the psychologist) [17]. In addition, there might be dentistry-specific barriers, such as a lack of knowledge about CBT methods and problems integrating workflows and new personnel (ie, CBT therapists) into the clinic.

### Internet-Based CBT

Internet-based CBT (ICBT) was developed to make CBT more accessible. ICBT is delivered over the internet through text, video clips, animations, and audio files instead of via a therapist in face-to-face sessions. ICBT uses the same mechanisms and principles as face-to-face CBT, and they have been shown to have comparable effect sizes [18]. Previous research has shown that ICBT, in which a therapist guides the patient, is associated with greater effects than unguided treatments [19]. Therapist-guided ICBT for fears and anxieties consists of a base of web-based material that conveys information and concrete exercises to reduce the patient’s fears, and the patient receives support from a psychologist through a web-based chat or email function. ICBT has been found to be effective for children with specific phobias [20,21] and children and adolescents with DP [22]. These studies have used parents as coaches guiding the child through the treatments.

The aim of this study was to test, in a randomized controlled trial (RCT), whether ICBT can reduce fear and increase willingness to receive dental treatment in children and adolescents with DP or IP.

## Methods

### Recruitment

Participants were recruited through dental clinics and social media advertisements that referred interested families to a website of the Department of Dental Medicine at Karolinska Institute. The study website contained brief information about the study and the targeted population. Interested parents (or caregivers) applied directly through the website and were then assigned a log-in for web-based screening. We informed dental clinics throughout Sweden and encouraged them to advertise for participants in the waiting rooms. We also asked dentists, especially specialists in pediatric dentistry, to recommend patients with DFA that was so severe that it interfered with dental treatment to apply to the study. In Sweden, both general and specialist pediatric dentistry are publicly funded and offered free of charge to all children. However, waiting times and the availability of specialist care differ between regions. Participants were recruited from October 2015 to December 2019. We planned to recruit 50 participants. However, owing to the slower-than-expected recruitment pace, we extended the originally planned recruitment time and ended the recruitment with 33 participants included in the study. Of the 58 interested applicants who provided informed consent and filled out the background information, 45% (26/58) were currently under treatment or on the waiting list of a specialist pediatric dentist. Furthermore, 76% (44/58) lived in the Stockholm region, whereas the rest were spread out throughout other regions of Sweden. Finally, 66% (38/58) were advised directly by their dentist to apply to the study, 2% (1/58) saw an advertisement on a notice board at their dentist’s office, 10% (6/58) found the study by searching on the internet or through social media advertisements, 3% (2/58) had heard of it through a friend or relative, and 19% (11/58) were patients in general dental care who were advised to apply to the study when consulting a specialist.

### Screening

The first step in web-based screening provided information on the study and details about informed consent from the caregivers (henceforth referred to as the parent or parents). After providing informed consent, the parent (parents had to designate one of them to fill out the questionnaires and conduct the telephone interview) and child provided basic background information on a questionnaire and then filled out 4 standardized questionnaires: the Picture-Guided Behavioral Avoidance Test (PG-BAT) [16], the Children’s Fear Survey Schedule–Dental Subscale (CFSS-DS) [23], the Children’s Negative Cognitions in Dentistry (CNCD) scale [22], and the Injection Phobia Scale for Children (IPSC) [24].

After assessment of the first screening step, we asked the parents of eligible participants to fill out the Development and Well-Being Assessment [25] and gave them new log-in credentials for accessing the survey on a different platform.

A clinical psychologist then interviewed the parent over the telephone. The semistructured diagnostic interview used the specific phobia section of the Kiddie Schedule for Affective Disorders and Schizophrenia for School-Age Children–Present and Lifetime (K-SADS-PL) [26] to establish whether the child had a specific phobia diagnosis of DP or IP. The interviewer also followed up the results of the Development and Well-Being Assessment with the parent when they indicated that the child might meet the potential exclusion criteria.

### Inclusion and Exclusion Criteria

Eligible participants who met the inclusion criteria (Textbox 1) were asked to join the study; they all agreed to participate.

Participants who met any of the exclusion criteria (Textbox 2) were excluded.

**Inclusion criteria.**

- The child’s age is between 8 and 15 years.
- Child *and* parents sign informed consent forms.
- A psychologist establishes a diagnosis of dental phobia or injection phobia according to the *Diagnostic and Statistical Manual of Mental Disorders (Fourth Edition)* from the results of the web-based parental version of the Development and Well-Being Assessment in the initial screening and of the Kiddie Schedule for Affective Disorders and Schizophrenia for School-Age Children–Present and Lifetime in the semistructured diagnostic interview.
- The Swedish language skills of the child *and* parents are sufficient to manage treatment and the questionnaires.
- Access to a computer and the internet is readily available.
- Child *and* parents have sufficient time and motivation to work with internet-based cognitive behavioral therapy 3 hours each week for 12 weeks.
- Parents agree to book at least 3 visits to the dental clinic during the 12 weeks of treatment.
- If the child is diagnosed with injection phobia, the parents agree to exposure training for intraoral injection phobia at the dental clinic even if the child does not need dental treatment.

**Exclusion criteria.**

- Full points on the child and parent versions of the Picture-Guided Behavioral Approach Test (the maximum score of 17 means that the child and the parent assess that the child is already able to manage the most challenging dental situations)
- A score of ≤31 on the child and parent versions of the Children’s Fear Survey Schedule–Dental Subscale and no diagnosis of injection phobia by the psychologist during screening
- A previously established diagnosis of a neurodevelopmental disorder or a likely diagnosis of a neurodevelopmental disorder according to the results of the Development and Well-Being Assessment or the psychologist during screening
- Other psychiatric disorders, such as, severe depression, eating disorders, or self-harm behavior, that require treatment first before dental-related specific phobias
- Current or planned psychiatric or psychological examination
- Current or planned psychological treatment
- Stressful life experiences in the previous 12 months, such as parental divorce or somatic illnesses, that the parent or psychologist consider an obstacle in treatment
- Cognitive behavioral treatment for dental fear and anxiety or dental phobia or injection phobia in the previous 3 years

### Randomization and Outcome Measures

**Figure 1:**
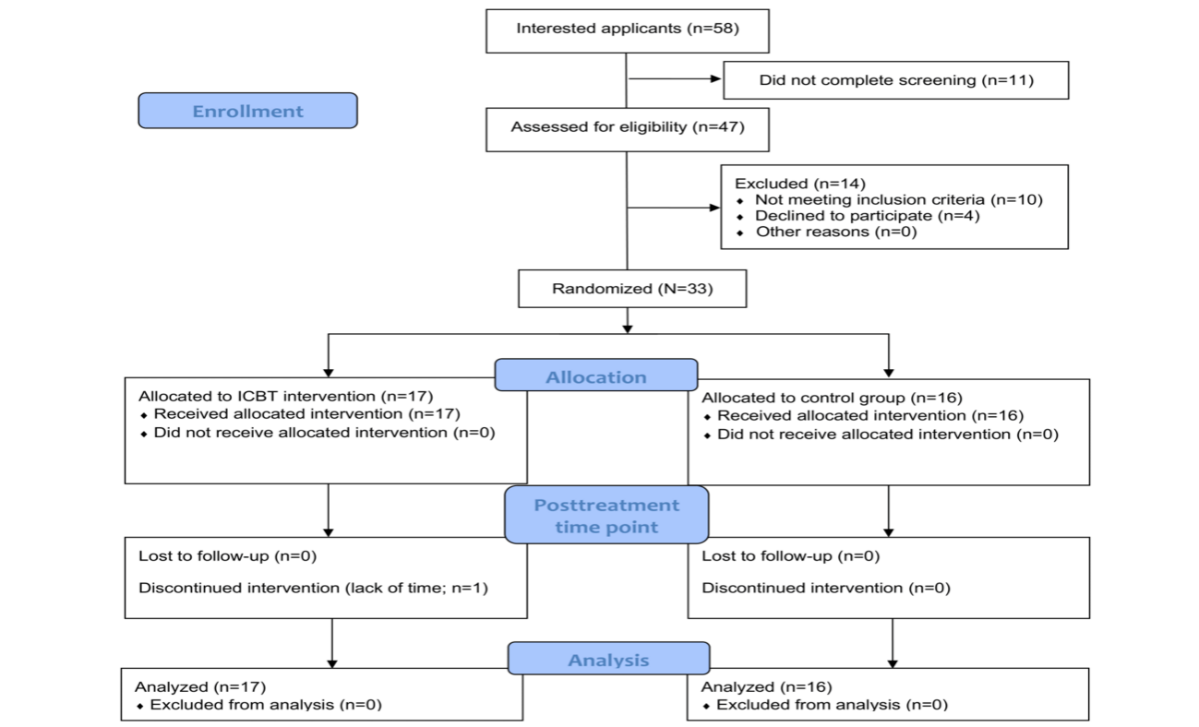
Flowchart of participant recruitment and allocation to the 2 study groups: internet-based cognitive behavioral therapy (ICBT) and control group. CONSORT: Consolidated Standards of Reporting Trials.

The study began by establishing a baseline for the outcome measures. These were the PG-BAT and CFSS-DS, which the child and parent rated separately. The CNCD scale, IPSC, and Self-Efficacy Questionnaire for Phobic Situations [27] were rated by the child only. Finally, the Parental Self-Efficacy Questionnaire for Dental Anxiety [22] was rated by the parent. One person uninvolved with the study and blinded to the identities of the participants then randomized the participants consecutively in a 1:1 ratio to active treatment or to a waiting list (the control group) using the list randomization tool [28] and the participants’ study ID number. New participants were continuously assigned using a randomized block design; the block size varied depending on the number of participants available at the time of randomization. In case there was 1 participant or an uneven number of participants, a dummy participant was added to the block to maintain a 1:1 ratio for randomization. A follow-up at 12 weeks, upon treatment completion, included all outcome measures in the baseline measurement in addition to a semistructured diagnostic interview with a psychologist over the telephone using the specific phobia section of the K-SADS-PL. Furthermore, the follow-up included a questionnaire concerning qualitative aspects of the child’s current dental anxiety and the ICBT treatment; response options included free text, multiple choice, and visual analog scales (VASs). The questionnaires also contained clear questions about adverse events or unwanted treatment effects, which the psychologists covered in their interviews. After the follow-up, the controls were offered the same treatment that the ICBT group had received. Figure 1 shows a flowchart of the participants in this study.

### Primary Outcome Measures

This study had 2 primary outcome measures. The first was the specific phobia section of the K-SADS-PL [26], which we used to diagnose DP and IP among the participants. A clinical psychologist included this in a semistructured interview over the telephone with the parent. The K-SADS-PL has been shown to be a reliable and valid instrument for assessing psychiatric diagnoses.

The second primary outcome measure was the PG-BAT [17,22], which the child and parent rated separately. The recommended outcome measure of CBT treatment for specific phobias is the Behavioral Approach Test [9,13]. The PG-BAT is similar and was used in a previous study of ICBT [22] for dental anxiety. The test has been shown to have good psychometric properties and measures the number of dental procedures (n=17; response options: yes=1 and no=0; Textbox 3) that the participants or parents felt that the participants could manage on their own. Pictures of 17 dental clinical procedures are organized according to increasing ability to provoke anxiety. Each image includes a written description of the situation and procedure. Scores range from 0 (not even able to enter the dentist’s room) to 17 (able to manage all treatment steps).

**The dental procedures in the Picture-Guided Behavioral Avoidance Test.**

- Enter the treatment room.
- Sit in the treatment chair with a paper bib around the neck.
- Sit in the treatment chair while the chair is lowered.
- Lie in the dental chair with the lamp turned on and the dental tools close by on a tray.
- Open the mouth and let the dentist look into the mouth.
- Let the dentist use a small saliva ejector in the mouth.
- Let the dentist blow air and water into the mouth.
- Undergo a clinical exam with a dental mirror.
- Undergo a clinical exam with a mirror and a dental probe.
- Let the dentist take an x-ray in the back of the mouth.
- Receive topical anesthesia.
- Receive an injection of a local anesthetic.
- Let the dentist use a large saliva ejector in the mouth.
- Let the dentist attach composite filling to the surface of a tooth.
- Let the dentist drill with a high-speed drill.
- Let the dentist polish a filling with a low-speed drill.
- Let the dentist extract a tooth.

### Secondary Outcome Measures

The CFSS-DS [22] measures DFA and comprises 15 items rated on a 5-point scale from *no fear* (1) to *high fear* (5). The items describe situations in dental and medical care and were rated separately by the child and the parent. The Swedish version of the CFSS-DS has been shown to have good psychometric properties [23].

The children rated the 5-item CNCD scale [22] on a 10-point VAS. The scale end points are 0=*no negative thoughts at all* and 1=*some negative thoughts*; happy and sad figures illustrate these concepts [10,14]. The questions regard the presence and strength of 5 negative thoughts that are common in dentistry: uncontrollability, distrust of dentists, unpredictability, dangerousness, and pain related to dentistry.

The IPSC [24] was rated by the child and consists of 18 items rated on a 5-point scale ranging from *no fear* (1) to *high fear* (5). The questions regard the fear of different situations and procedures related to injections. This test has been shown to have good psychometric properties [24].

Participants rated the Swedish translation of the 14-item Self-Efficacy Questionnaire for Phobic Situations [27] on a 5-point scale with the following end points: 1=*low self-efficacy* and 5=*high self-efficacy*.

Parents rated a Swedish version of the 12-item Parental Self-Efficacy Questionnaire for Dental Anxiety [22] on a 10-point scale with the following end points: 0=*no parental self-efficacy* and 10=*very high parental self-efficacy*. The instrument was evaluated in our open trial study on ICBT for dental anxiety in children and adolescents [22].

### The ICBT Intervention Platform

The form of ICBT used in this study was based on a previously published manual [17] that was used in a pilot study [22]. The central component of the treatment was exposure through video and audio recordings, a toolkit for use at home, and in vivo exposure at the dental clinic. Other components of the treatment were psychoeducation, behavioral analyses, controlled breathing, and parental education (Textbox 4). Parents were asked to decide among themselves who would be responsible for coaching and supporting the children and adolescents, thereby becoming the designated coach throughout the treatment. The second treatment module targeted the coach with information on how to support, motivate, and assist the children and adolescents with their assignments.

**Outline of the 12 internet-based cognitive behavioral therapy (ICBT) modules (one module each week).**

- Introduction to cognitive behavioral therapy and the web-based treatment
- Psychoeducation, practical arrangements, home assignment, how to guide a child to elicit and reinforce behavior change, reward strategies, and enhancing the child’s self-efficacy (for the coach only)
- Behavioral analyses, child psychoeducation and treatment rationale, and goal setting
- Constructing an exposure list and beginning exposure
- Continued exposure (films and training package) and controlled breathing
- Dentistry-related communication training and preparation for the first dental visit
- Evaluation of the dental visit and cognitive restructuring
- Midterm evaluation of ICBT and of exposure at the dentist and relaxation techniques
- Pain and pain management education; fear, thoughts, and pain; and focus shift and acceptance training
- Problem-solving and mindfulness training
- Repetition, strategies for maintaining change and relapse prevention, and letter to yourself
- Relapse prevention plan, enhance your self-efficacy, and diploma

The intervention and all data collection occurred on a secure web-based platform hosted by the Internetpsykiatri (internet psychiatry) unit run by Stockholm Health Care Services, Region Stockholm, Sweden. The coaches and children and adolescents shared the same log-in. Each module indicated who was responsible for completing the various measurements, treatment modules, and assignments. One of the assignments for the coach, for example, was to call the participant’s local dental clinic and book at least 3 visits for in vivo exposure, which the dental staff would perform. Participants downloaded information and instructions on exposure from the platform and mailed printed or digital copies of these to the clinic. During week 2, the participants received a toolkit with dental instruments and a VAS that the children and adolescents and coach could use for the exposure assignments at home (Multimedia Appendix 1).

The ICBT intervention comprised 12 web-based treatment modules that were made accessible to the participants, with 1 module per week. Each participant was assigned a psychologist who supported them throughout the treatment. In total, 3 psychologists were involved in this study; all had a 5-year degree in clinical psychology at a minimum. In addition, all 3 had face-to-face clinical dental experience in CBT with children and adolescents who had DP or IP. All modules had some kind of text; most modules also had pictures, animations, and videos and audio of various dental procedures for exposure purposes. Multimedia Appendices 2-4 show example screenshots of the interface and treatment modules.

Each week, the children and adolescents and coaches completed a new module that concluded with an assignment containing knowledge questions about the module and practical exercises. Each week, participants sent their responses to the questions and a log of their assignments to their psychologist, who would send feedback and grant access to the next module within 2 days. Participants could also message their psychologist directly through their account on the platform and expect a reply, on average, within 2 working days. The psychologists would send reminders to inactive participants about continuing work with the modules via SMS text message and email. If participants were inactive for >10 days, the psychologist would try to reach them by telephone.

A total of 12 weeks after treatment had begun, and regardless of whether the participants had finished the treatment modules, all treatment modules were made available on the platform. The account of the children and adolescents and coaches with its log-in credentials remained active for the next 12 months; however, participants were no longer able to communicate with their psychologist.

### Statistical Analysis

All statistical analyses were conducted using SPSS (version 27; IBM Corp). We accepted a 5% type-I error in all analyses. To compare the means between the 2 study conditions, we used 2-tailed *t* tests. Repeated-measure ANOVAs were conducted to evaluate possible differences in changes over the 12 weeks (baseline to follow-up) between the ICBT and control groups. We estimated the effect size using the Cohen *d*, that is, the standardized mean difference [29]. Chi-square tests of independence were conducted to evaluate possible between-group differences in meeting the diagnostic criteria. Before conducting our analyses, we checked the data for normality. We expected the effects to be in line with our open trial study using the same procedures and treatment [22]. Thus, the power calculation was based on an estimated effect size of Cohen *d*=1.0 and showed that, to obtain 80% power (Cronbach α=.05), 17 participants in each arm (N=34) were required. We used an intention-to-treat design; that is, participants were included in the analyses irrespective of the extent to which they had completed the treatment.

### Ethical Considerations

Before the participants could complete screening and enter the study, informed consent was obtained. Information about the study and informed consent was provided in Swedish and included permission for secondary analysis of the data without additional consent. Informed consent was provided by both caregivers separately if there were 2. There was no compensation given to the participants, and similar to all dental care for children and adolescents in Sweden, they received the intervention for free. For added security, the secure web-based platform in which data were collected required a 2-step authentication via SMS text message for logging in, both for participants and the study staff. When extracting data from the platform for analysis, they were deidentified, and an anonymous study ID was used as an identifier for each participant. The regional ethics review board of Stockholm approved this study (ID 2014/633-31/5).

## Results

### Overview

All participants had a diagnosis of DP or IP (or both) at baseline. Of the 33 children included in this study, 21 (64%) were female. The mean age of the study sample was 11.2 (SD 1.9) years. During previous dental treatment, sedation or general anesthesia had been administered or restraint had been necessary for all but 2 participants (31/33, 94%; 1 participant in each group). Table 1 presents the participant characteristics.

Table 2 presents the baseline scores of the children and their parents in the 2 study groups. The 2-tailed *t* tests revealed no significant between-group differences in these measures (all *P*>.05). Chi-square tests of independence showed no significant between-group associations in meeting the diagnostic criteria for DP, IP, or DP and IP (all *P*>.05).

**Table 1:** Participant characteristics of the internet-based cognitive behavioral therapy (ICBT) and control groups (N=33).

Variable		ICBT (n=17)	Control (n=16)	Total (N-=33)
Age (y), mean (SD)		11.3 (1.8)	11.1 (1.9)	11.2 (1.9)
Female participants, n (%)		10 (59)	11 (69)	21 (64)
Both parents born in Sweden, n (%)		15 (88)	14 (88)	29 (88)
Living with both parents, n (%)		16 (94)	12 (75)	28 (85)
**Duration (mo), mean (SD)**				
	Fear of the dentist	38 (30)	47 (50)	45 (41.5)
	Fear of injections	60 (41)	72 (55)	69.8 (48.4)
**Diagnosis, n (%)**				
	Dental phobia	14 (82)	14 (88)	28 (85)
	Injection phobia	15 (88)	14 (88)	29 (88)
	Dental and injection phobia	12 (71)	12 (75)	24 (73)
	Another specific phobia^a^	5 (29)	5 (31)	10 (30)
**The following occurred during previous dental treatment, n (%)**				
	Sedation was administered	16 (94)	15 (94)	31 (94)
	Restraint was necessary	10 (59)	8 (50)	18 (55)
	General anesthesia was administered	4 (24)	2 (12)	6 (18)

^a^Diagnosis of a specific phobia other than dental or injection phobia.

**Table 2:** Baseline self-report scores of the children and their parents in the internet-based cognitive behavioral therapy (ICBT) and control groups.

Outcome variable			ICBT (n=17), mean (SD; 95% CI)		Control (n=16), mean (SD; 95% CI)		*P* value^a^
**Child**							
	Dental procedures managed^b^	11.1 (4.2; 9.0-13.3)		11.7 (3.2; 10.0-13.4)		.67	
	Dental fear and anxiety^c^	37.1 (10.5; 31.6-42.5)		35.3 (12.3; 28.7-41.8)		.65	
	Negative cognitions^d^	25.5 (13.0; 18.8-32.2)		22.0 (11.6; 15.8-28.2)		.43	
	Injection fear^e^	41.7 (10.5; 36.2-47.1)		43.3 (12.1; 36.9-49.7)		.68	
	Self-efficacy^f^	25.6 (6.6; 22.2-29.0)		29.8 (7.3; 26.0-33.7)		.09	
**Parent**							
	Dental procedures managed^b^	10.6 (4.6; 8.2-13.0)		11.2 (4.1; 9.0-13.4)		.70	
	Dental fear and anxiety^c^	35.5 (9.5; 30.6-40.4)		36.8 (9.7; 31.7-42.0)		.69	
	Parental self-efficacy^g^	111.2 (15.2; 103.4-119.0)		105.6 (18.3; 95.9-115.4)		.34	

^a^*P* values are based on the 2-tailed *t* test.

^b^Picture-Guided Behavioral Avoidance Test; score range: 0 to 17.

^c^Children’s Fear and Survey Schedule–Dental Subscale; score range: 15 to 75.

^d^Children’s Negative Cognitions in Dentistry scale; score range: 0 to 50.

^e^Injection Phobia Scale for Children; score range: 18 to 90.

^f^Self-Efficacy Questionnaire for Phobic Situations; score range: 14 to 70.

^g^Parental Self-Efficacy Questionnaire for Dental Anxiety; score range: 0 to 120.

### Treatment Adherence and Dropout Frequency

All participants underwent the 12-week follow-up; thus, no data were missing. Of the 17 participants in the ICBT group, 1 (6%) chose not to continue the intervention after week 2 because of other priorities and lack of time; we categorized this participant as a dropout. The other 94% (16/17) of the participants completed at least 5 modules and started the first steps of exposure training. A total of 81% (13/16) of the participants were considered treatment completers as they finished the most important steps of the treatment (module 8) and were conducting in vivo exposure sessions at their local dental clinic at the time of the follow-up. The mean number of completed modules after 12 weeks was 8.4 (SD 3.4). In total, 12% (2/16) of the participants in the control group chose not to enroll in ICBT after the study was concluded.

### Primary Outcome Measures

In the ICBT group, 41% (7/17) of the participants no longer met the diagnostic criteria for DP, IP, or DP and IP at the posttreatment clinical interview compared with 0% in the control group. A chi-square test of independence showed that the difference was significant (χ^2^_1_=8.4; *P*=.004). A total of 65% (11/17) of the participants lost at least 1 of their earlier diagnoses of either DP or IP (N=33, χ^2^_1_=15.5; *P*<.001) compared with 0% in the control group. Table 3 presents the results of the intervention stratified by diagnosis.

**Figure 2:**
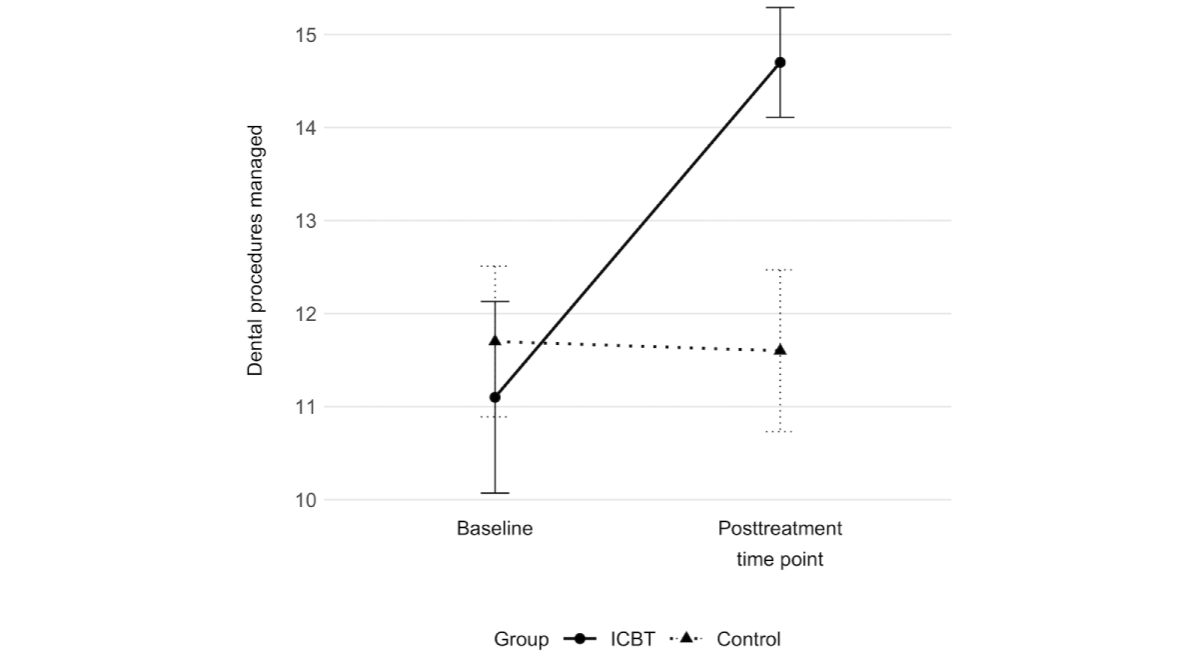
Number of dental procedures managed according to the Picture-Guided Behavioral Avoidance Test (PG-BAT). Child ratings at 2 time points (baseline and posttreatment time point) in the 2 study groups: internet-based cognitive behavioral therapy (ICBT) and control group.

**Figure 3:**
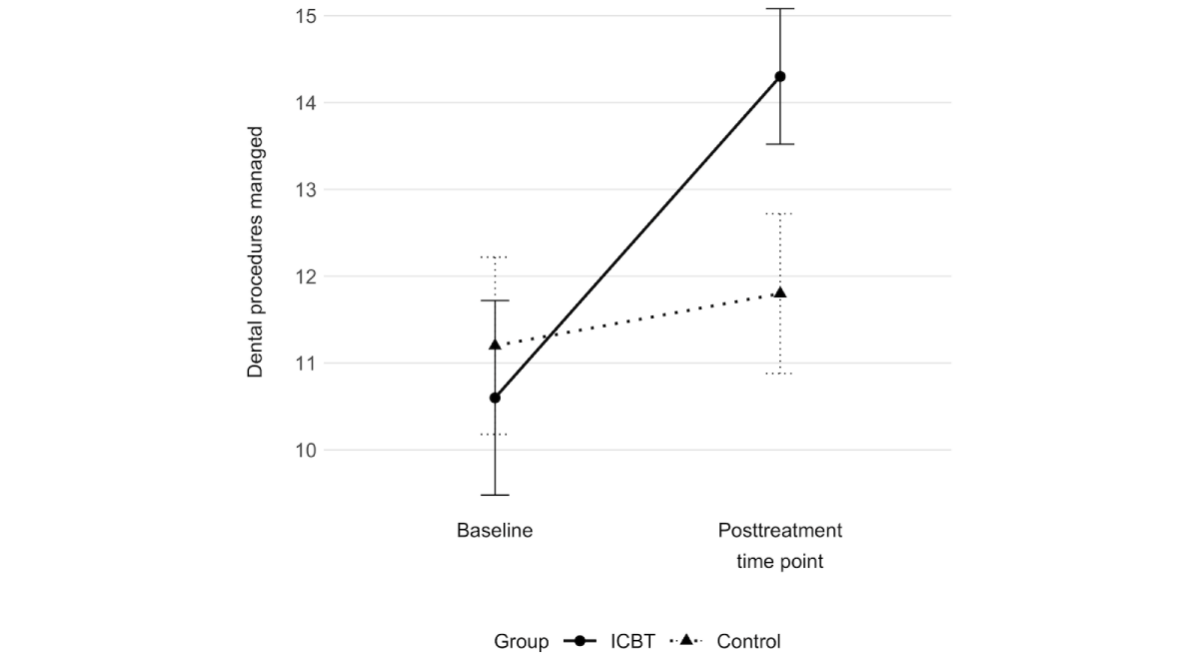
Number of dental procedures managed according to the Picture-Guided Behavioral Avoidance Test (PG-BAT). Parent ratings at 2 time points (baseline and posttreatment time point) in the 2 study groups: internet-based cognitive behavioral therapy (ICBT) and control group.

Furthermore, children in the ICBT group who rated the PG-BAT noted being able to manage a significantly larger number of dental procedures at the follow-up (mean 3.6, SD 3.1) compared with children in the control group, who reported a decrease over the same period (mean −0.1, SD 1.4; *P*<.001; Figure 2). Parents in the ICBT group also noted that their children were able to manage a significantly larger number of dental procedures (mean 3.7, SD 3.7) compared with parents in the control group (mean 0.6, SD 2.5; *P*=.009; Figure 3). The between-group effect sizes (Cohen *d*) at the follow-up, calculated from the group-by-time interaction effects resulting from a repeated-measure ANOVA analysis of the child- and parent-rated PG-BAT, were 1.6 (95% CI 0.8-2.3) and 1.0 (95% CI 0.3-1.7), respectively; this indicated large treatment effects in the primary outcome measuring willingness and capability to manage dental treatment.

**Table 3:** Participants meeting the criteria for diagnoses of dental phobia or injection phobia at baseline and at the follow-up in the internet-based cognitive behavioral therapy (ICBT) and control groups.

Diagnosis	Treatment period, n (%)						Chi-square (*df*)			*P* value
	ICBT group (n=17)		Control group (n=16)							
	Before	After	Before	After						
Dental phobia	14 (82)	7 (41)	14 (88)	14 (88)	9.4 (1)			.002		
Injection phobia	15 (88)	10 (59)	14 (88)	14 (88)	5.6 (1)			.02		

### Secondary Outcome Measures

During the 12 weeks between baseline and follow-up, significantly larger improvements were observed in the ICBT group than among the controls in all secondary outcome measures except parental self-efficacy (Table 4) for mean changes and the group-by-time interaction effects calculated in the repeated-measure ANOVA. Multimedia Appendix 5 presents a complete table of means and SDs at follow-up for all outcome measurements stratified by allocation group. Multimedia Appendix 6 presents within-group effects for the ICBT group; no within-group effects were observed in the control group. Participants reported no adverse events or unintended effects during the intervention.

**Table 4:** Child- and parent-rated changes between baseline and follow-up in secondary outcomes and between-group effect sizes in the internet-based cognitive behavioral therapy (ICBT) and control groups.

Participant and change			ICBT group (n=17)					Control group (n=16)					Repeated-measure ANOVA^a^		
			Participants, n (%)		Values, mean (SD)		Participants, n (%)			Values, mean (SD)		*P* value^b^			Cohen *d* (95% CI)
**Child**															
	Δ Dental fear and anxiety^c^	17 (100)		−11.7 (8.7)		16 (100)			2.6 (9.2)		*<.001* ^d^			−1.6 (−2.4 to −0.8)	
	Δ Negative cognitions^e^	15 (88)		−13.3 (9.7)		16 (100)			1.7 (13.1)		*.001*			−1.3 (−2.1 to −0.5)	
	Δ Injection fear^f^	16 (94)		−10.6 (10.0)		16 (100)			0.7 (13.4)		*.01*			−1.0 (−1.7 to −0.2)	
	Δ Self-efficacy^g^	16 (94)		18.3 (12.2)		16 (100)			−0.3 (5.8)		*<.001*			2.0 (1.1 to 2.8)	
**Parent**															
	Δ Dental fear and anxiety^c^	17 (100)		−10.88 (9.38)		16 (100)			0.9 (8.9)		*.001*			−1.3 (−2.0 to −0.5)	
	Δ Parental self-efficacy^h^	17 (100)		3.71 (13.68)		16 (100)			−3.8 (8.2)		.07			0.7 (−0.1 to 1.4)	

^a^Group-by-time interaction effects.

^b^*P* values are based on group-by-time interaction effects of repeated-measure ANOVA.

^c^Children’s Fear and Survey Schedule–Dental Subscale; score range: 15 to 75.

^d^Italicized values indicate significance.

^e^Children’s Negative Cognitions in Dentistry scale; score range: 0 to 50.

^f^Injection Phobia Scale for Children; score range: 18 to 90.

^g^Self-Efficacy Questionnaire for Phobic Situations; score range: 14 to 70.

^h^Parental Self-Efficacy Questionnaire for Dental Anxiety; score range: 0 to 120.

## Discussion

### Principal Findings

After treatment, 41% (7/17) of the participants in the ICBT group no longer met the diagnostic criteria for either DP or IP, whereas all participants in the control group did. Furthermore, an additional 24% (4/17) of children in the ICBT group no longer met the diagnostic criteria for one of their 2 diagnoses at baseline, totaling 65% (11/17) of children in the ICBT group in whom at least one diagnosis had remitted. Compared with the control group, participants in the ICBT group could also willingly receive significantly more treatment steps at the dentist, and their fear, anxiety, and negative cognitions toward dentistry and injections significantly decreased after 12 weeks. As rated by both the children and parents, the effect size calculated from the primary outcome (PG-BAT) was large. In addition, children in the ICBT group reported higher self-efficacy. In summary, this RCT indicates that ICBT is an effective treatment for children with DP and IP.

### Comparison With Prior Work

This is the first RCT of ICBT for DP and IP in pediatric dentistry. The effect sizes are in line with those of earlier studies on in-person exposure-based CBT methods in pediatric dentistry. Our research group previously conducted a nonrandomized pilot study using the same interventions as in this trial [22]. That study found within-group effect sizes between baseline and follow-up of 1.5 (95% CI 0.7-2.3) for the child-rated PG-BAT and 1.0 (95% CI 0.5-1.6) for the child-rated CFSS-DS; these are comparable with the within-group effect sizes in this study, which were 1.2 (95% CI 0.5-1.8) and 1.3 (95% CI 0.7-2.0), respectively. In the previous pilot study [22], ICBT participants were further improved at the 1-year follow-up, suggesting that the results of CBT for DP and IP are stable and can even increase over time.

An RCT of face-to-face CBT for dental anxiety in children and adolescents found strong between-group effects of Cohen *d*=1.4 after treatment and 1.9 at the 1-year follow-up for the main outcome of a clinician-administered Behavioral Approach Test [15]. After treatment, 64% of the patients in the CBT group no longer met the diagnostic criteria of their initial diagnosis, and at the 1-year follow-up, the proportion was 91%. This study used an active control group receiving care from specialist pediatric dentists, indicating that exposure-based CBT can yield improvements above and beyond those achieved with traditional care at specialist dental clinics for pediatric care. When calculated from the child-rated CFSS-DS, the within-group effect size of that study was 1.8 (95% CI 0.9-2.8) compared with a within-group effect size of 1.3 (95% CI 0.7-2.0) for the ICBT group in this study.

Finally, a research group in Norway conducted a 5-session CBT series for children and adolescents diagnosed with intraoral IP [16]. In that study, dentists specially trained in CBT carried out treatment, showing that exposure-based CBT can also be effectively implemented by dentists without the need for psychologists. During CBT treatment, 70% (47/67) of the participants were able to tolerate an injection; at the 1-year follow-up, 69% (34/49) were able to manage the required intraoral injections by their regular dentist according to dental records. As no effect sizes or correlations were reported, we calculated the Cohen *d* of the CFSS-DS from the pre- and posttreatment scores—33.8 (SD 9.7) and 23.9 (SD 6.7)—of the sample (N=57). The resulting effect size of 1.2 (95% CI 0.6-1.8) is once again comparable with the within-group effects of 1.3 (95% CI 0.7-2.0) for the ICBT group in this study.

Taken together, there is an emerging base of evidence for exposure-based CBT in pediatric dentistry, and it seems that this type of treatment can be delivered effectively in several ways. An important avenue for future research is to investigate whether ICBT also produces effects superior to those of active control conditions and noninferiority compared with traditional face-to-face CBT.

### Clinical Implications

One of the criteria for a phobic diagnosis is that it interferes significantly with a person’s normal functioning. In the case of DP and IP, this usually means not being able to receive needed dental care, negatively affecting the person’s dental and general health [17]. This study shows that ICBT can be of major clinical value in dentistry. ICBT lowers avoidance and fear, enabling children and adolescents to willingly receive dental care without the need for sedation or restraint methods; thus, the dental and general health of children and adolescents who previously avoided dental care because of DP or IP will improve over the long term.

An important clinical aspect of this study was that dental treatment and in vivo exposure were provided by general dental personnel, many times dental assistants, with most not having any association with the research team and having no previous education in the method or specialist training. Earlier studies have also used exposure-based CBT provided by trained dental personnel [11,12,16] face-to-face. This requires a shift in roles from their traditional dental practices to providing exposure therapy, with communication style and time viewed as the most important factors for the treatment to be successful [30]. However, our results suggest that, when ICBT is implemented, it is clinically feasible to only briefly instruct the dentist on how to conduct exposure with patients and their parents, and this information can then be conveyed to the dental assistant and implemented sufficiently well from a therapeutic perspective. We consider this important as it indicates that exposure-based ICBT can be integrated into routine dental care practices and, thus, has the potential to be effectively disseminated while also requiring less time invested by the dental personnel as important parts of the treatment are provided on the web-based platform. In addition, participants reported no adverse events, indicating that ICBT is safe even when in vivo exposure is conducted by general dental personnel with no training in CBT and at home by the patients and their parents themselves.

Previous research has indicated a need for greater use of evidence-based CBT methods with children and adolescents in dentistry [13]. ICBT has been shown to be as effective as face-to-face treatment in many settings and for different conditions [18]. More specifically, ICBT has been shown to be effective for specific phobias [20]. This study showed that ICBT is also an effective treatment that can be used for children with DP or IP in dentistry. As no other evidence-based treatment for children or adolescents with DP or IP in dentistry currently exists, ICBT could fill a gap by providing an evidence-based treatment for the field of dentistry if disseminated correctly. ICBT can potentially be administered to more patients at a lower cost, thus overcoming the hurdle of too few CBT-trained clinicians. Furthermore, ICBT is more accessible than face-to-face CBT for patients as ICBT allows them to freely choose treatment times. This also extends to adult populations. Currently, no trials of ICBT for adults have been conducted, but we find it highly probable that ICBT for adults will be as effective as it is for children and adolescents.

Dentists may hesitate to recommend CBT treatment to their patients, which the slow recruitment in this study suggests. Previous studies have also shown that recruitment for CBT trials in dentistry is difficult [31]. This might be partly owing to a lack of interest by general practicing dentists to refer patients for CBT, which demonstrates the need to make CBT treatment attractive to dental clinicians. Consequently, one aspect that we believe is key for successful dissemination of ICBT is to increase interest in general dentistry for this type of treatment; in this way, families can be referred to and recommended exposure-based treatments when motivation is likely to be high (ie, shortly after a dental visit in which the child expressed marked fear).

Finally, this study excluded participants with neurodevelopmental disorders, a group of patients that is common in specialist pediatric dentistry. Future research needs to explore how exposure-based treatments can be successfully adapted for these patients.

### Strengths and Limitations

Important strengths of this study were the randomized controlled design, absence of missing data, low treatment dropout rate and generally high treatment adherence, and use of both validated self-rating scales and clinician assessments, which addressed methodological problems observed in previous research [1]. This study also had high external validity as the dental treatment and in vivo exposure were administered at general dental clinics by personnel with no formal training in CBT. Finally, the sample was clinically relevant, having had fears of visiting the dentist for an extended time and previous experience of unsuccessful treatment to reduce their DFA or DP and IP through sedation, restraint, or general anesthesia.

Regarding limitations, the control group in this study was a waiting list. We initially hoped to establish the efficacy of ICBT treatment before testing it against active control conditions or establish noninferiority by comparing it with face-to-face CBT. The blinding of the clinicians who performed the follow-up interview was not assessed in this study. In addition, the study had a fairly small sample size, which precludes, for example, meaningful subgroup analyses. Furthermore, we used the IP diagnosis instead of intraoral IP in this study. Although it was required that the IP affect the dental treatment of participants when entering the study, it would have been preferable to use the intraoral IP diagnosis. This is because, in some cases, after the treatment, we had patients who no longer met the criteria for intraoral IP diagnosis but still met the criteria for IP diagnosis (ie, being able to receive intraoral injections in dentistry but not intramuscular vaccine injections). However, this only attenuated our results, which would have been even stronger if an intraoral IP diagnosis had been used.

Another limitation of this study is that recruitment through advertisements might not reach the general population, creating a nonrepresentative group. This could be another factor behind the slow recruitment, suggesting treatment barriers for certain groups that warrant further investigation. Speculatively, some families may have too few resources or be reluctant to commit to a program as long as 12 weeks. This is further exemplified by one participant in the study who dropped out because of time constraints and 3 participants who did not complete the modules during the treatment time. The extra burden of investing time in a treatment of this type makes it unsuitable for some families and individuals. This limits the use of both CBT and ICBT in its current form, and more research is needed on how to provide a feasible, evidence-based alternative for this group. Perhaps one alternative that should be tested is an even shorter course in exposure-based CBT that is less text dependent.

Finally, this study was conducted in Sweden, which has publicly funded, free-of-charge dental services for children. The generalizability of the findings to other dental health care contexts needs to be further investigated. More specifically, how the cost of this type of treatment might influence its acceptability for the patient and dentist in different contexts needs to be explored.

### Conclusions

ICBT for DP or IP seems to be an effective treatment for children and adolescents. This therapy reduces fear and enables the child to willingly receive dental treatment, thereby leading to improved dental and general health. ICBT should be considered a method that increases accessibility to effective psychological treatment in pediatric dentistry.

## Appendix

Multimedia Appendix 1 Content and photographs of the exposure toolkit and visual analog scale.

Multimedia Appendix 2 Screenshot from internet-based cognitive behavioral therapy (module with text).

Multimedia Appendix 3 Screenshot from internet-based cognitive behavioral therapy (module with text and picture).

Multimedia Appendix 4 Screenshot from internet-based cognitive behavioral therapy (home assignment at end of module).

Multimedia Appendix 5 Scores of the children and their parents in the internet-based cognitive behavioral therapy (n=17) and control (n=16) groups at the follow-up.

Multimedia Appendix 6 Within-group changes and effect sizes between baseline and treatment follow-up for the internet-based cognitive behavioral therapy group.

Multimedia Appendix 7 CONSORT-eHEALTH checklist (V 1.6.1).

## Abbreviations

CBT: cognitive behavioral therapy
CFSS-DS: Children’s Fear Survey Schedule–Dental Subscale
CNCD: Children’s Negative Cognitions in Dentistry
DFA: dental fear and anxiety
DP: dental phobia
ICBT: internet-based cognitive behavioral therapy
IP: injection phobia
IPSC: Injection Phobia Scale for Children
K-SADS-PL: Kiddie Schedule for Affective Disorders and Schizophrenia for School-Age Children–Present and Lifetime
PG-BAT: Picture-Guided Behavioral Avoidance Test
RCT: randomized controlled trial
VAS: visual analog scale

## Data Availability

The data sets generated and analyzed during this study are available from the corresponding author upon reasonable request.
