# Supplementary material for: Internet-Based Cognitive Behavioral Therapy for Children and Adolescents With Dental or Injection Phobia: Randomized Controlled Trial
Source: J Med Internet Res. 2024 Feb 21;26:e42322. doi: 10.2196/42322 (PMC10918554; doi:10.2196/42322)
Supplement: Multimedia Appendix 1 [file jmir_v26i1e42322_app1.docx]

| 1. Dental exam gloves in various sizes (XS, S, M, L) 2. Face mask 3. Spiral suction 4. Cotton rolls (8) 5. Cotton swabs (4) 6. Syringe needles (2) 7. A reflecting key ring used as reward 8. Mouth mirror 9. Toothbrush 10. Suction aspirator, flexible handle 11. Saliva ejector 12. Probe 13. X-ray plate, in plastic with packaging foam 14. Tooth dryer 15. Topical numbing gel: Lidocaine APL 5% 16. Plastic cup 17. Visual Analogue Scale (VAS) |
| --- |


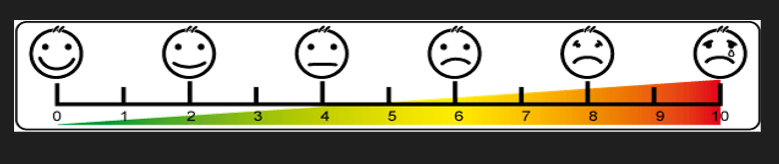


Item #17 from the list of Supplement 1. Contents and photo of the exposure toolkit and Visual Analogue Scale (VAS).
