# Supplementary figures and images for: Internet-Based Cognitive Behavioral Therapy for Children and Adolescents With Dental or Injection Phobia: Randomized Controlled Trial
Source: J Med Internet Res. 2024 Feb 21;26:e42322. doi: 10.2196/42322 (PMC10918554; doi:10.2196/42322)

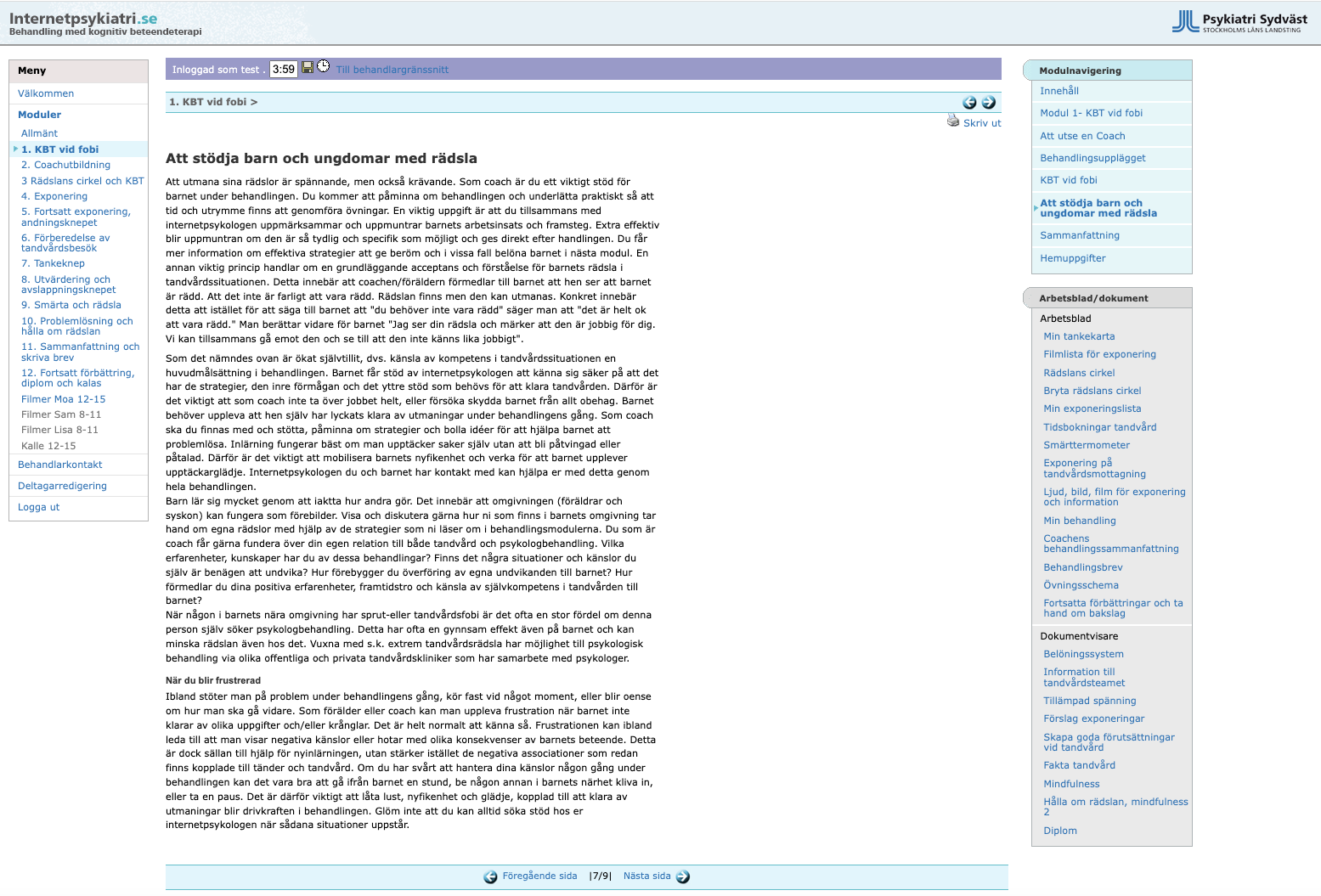

Supplement: Multimedia Appendix 2 [file jmir_v26i1e42322_app2.png]

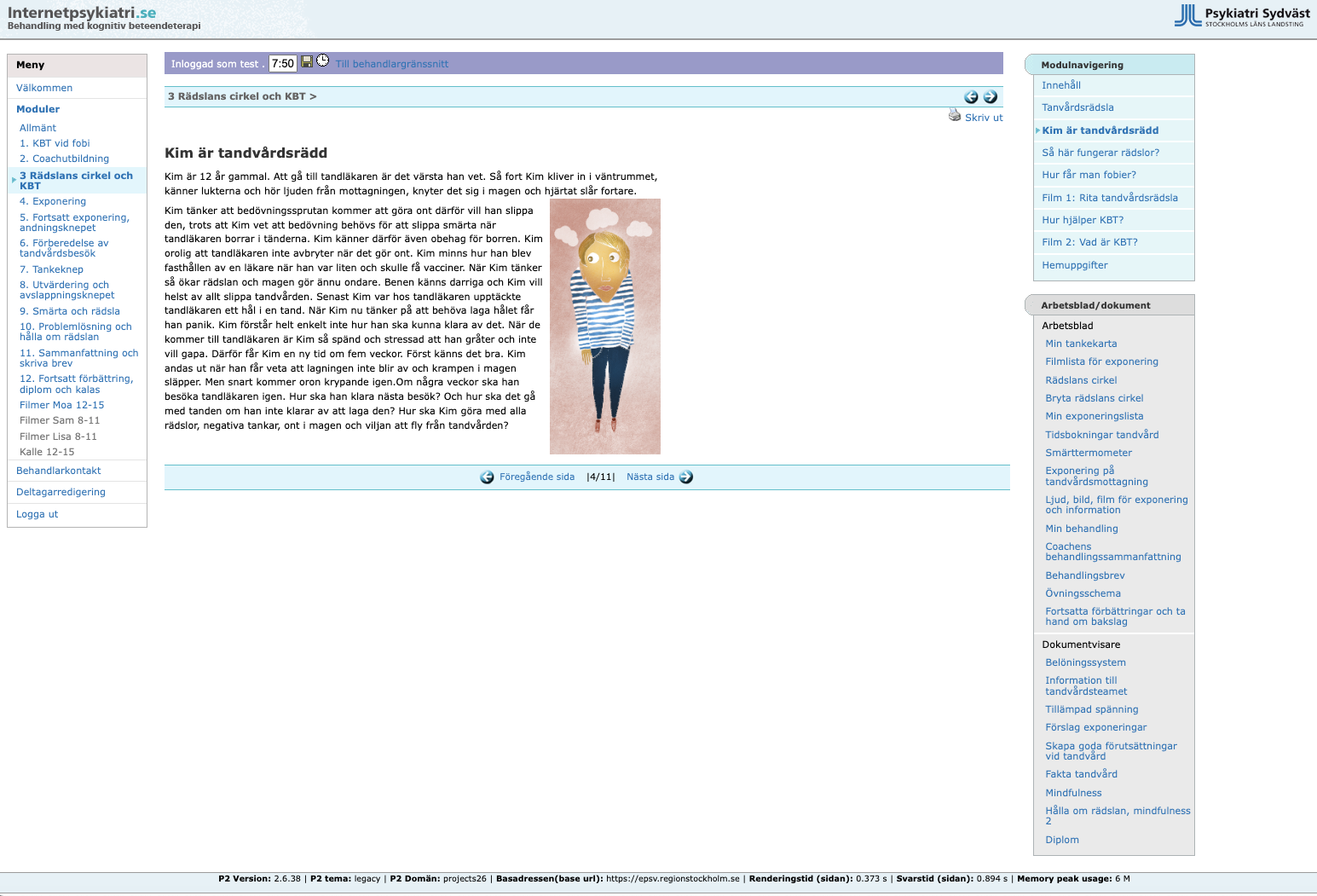

Supplement: Multimedia Appendix 3 [file jmir_v26i1e42322_app3.png]

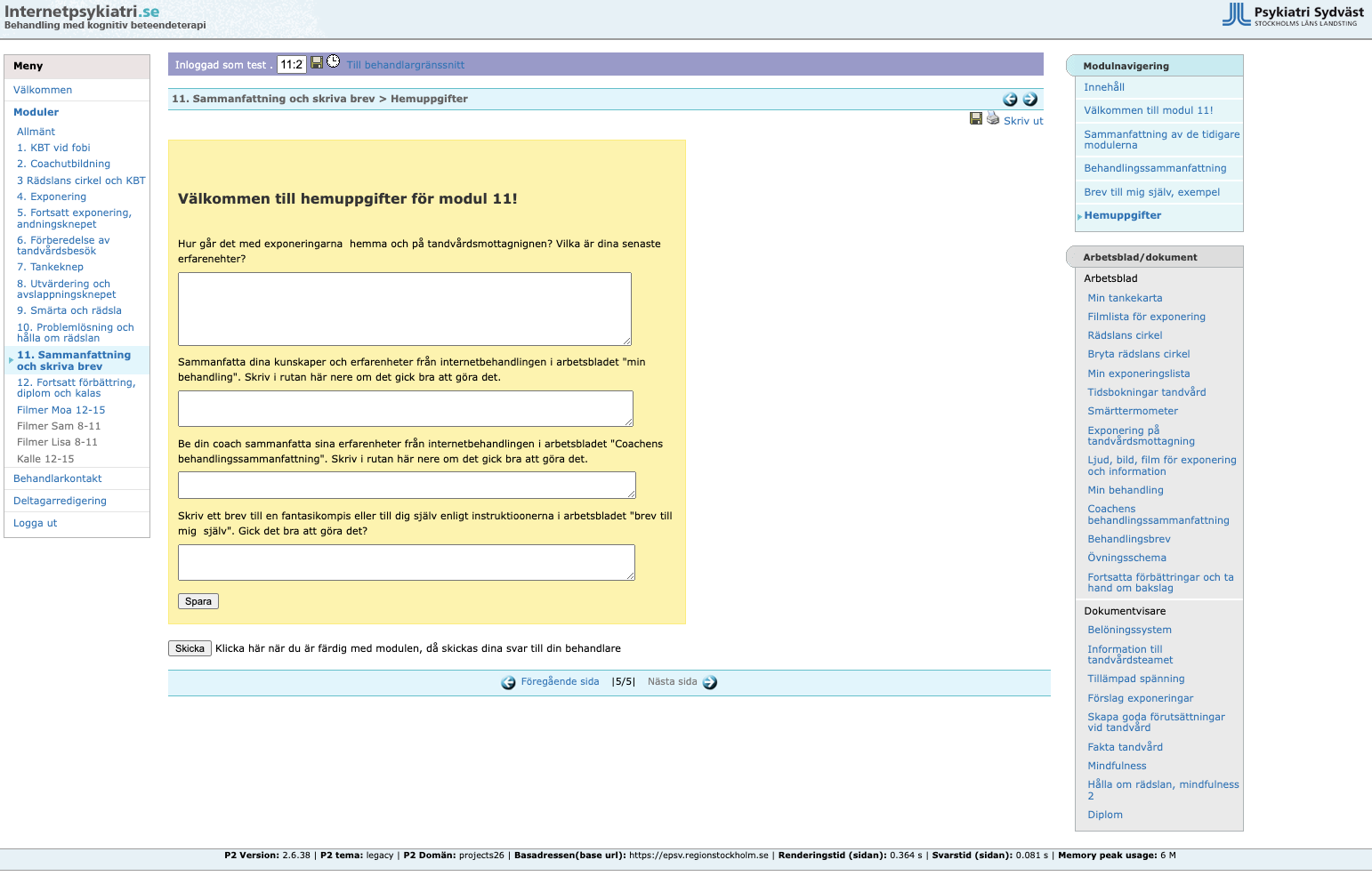

Supplement: Multimedia Appendix 4 [file jmir_v26i1e42322_app4.png]
