# Supplementary material for: Internet-Based Cognitive Behavioral Therapy for Children and Adolescents With Dental or Injection Phobia: Randomized Controlled Trial
Source: J Med Internet Res. 2024 Feb 21;26:e42322. doi: 10.2196/42322 (PMC10918554; doi:10.2196/42322)
Supplement: Multimedia Appendix 5 [file jmir_v26i1e42322_app5.docx]

| Outcome variable | ICBT group  Mean (SD) | Control group  Mean (SD) | Mean difference | 95% CI | *P* value^a^ |
| --- | --- | --- | --- | --- | --- |
| *Child* |  |  |  |  |  |
| Dental procedures managed^b^, n | 14.7 (2.4) | 11.6 (3.5) | 3.1 | 1.0 to 5.3 | **.005** |
| Dental fear and anxiety^c^ | 25.4 (7.7) | 37.8 (14.9) | –12.4 | –20.7 to –4.1 | **.005** |
| Negative cognitions^d^ | 12.1 (12.4) | 23.7 (13.7) | –11.6 | –21.2 to –1.9 | **.020** |
| Injection fear^e^ | 31.4 (8.3) | 44.0 (13.1) | –12.6 | –20.5 to –4.7 | **.003** |
| Self-efficacy^f^ | 44.0 (15.4) | 29.5 (7.1) | 14.4 | 5.8 to 23.1 | **.002** |
| *Parent* |  |  |  |  |  |
| Dental procedures managed^b^, n | 14.3 (3.2) | 11.8 (3.7) | 2.5 | 0.1 to 5.0 | **.042** |
| Dental fear and anxiety^c^ | 24.6 (8.0) | 37.7 (13.5) | –13.1 | –21.0 to –5.3 | **.002** |
| Parental self-efficacy^g^ | 115.0 (22.1) | 101.8 (22.0) | 13.1 | –2.5 to 28.8 | .097 |

^a^P values are based on *t* test

^b^The picture-guided behavioral avoidance test (PG-BAT); score range: 0-17

^c^The Children’s Fear and Survey Schedule – Dental Subscale (CFSS-DS); score range: 15-75

^d^The Children’s Negative Cognitions in Dentistry (CNCD) scale; score range: 0-50

^e^The Injection Phobia Scale for Children (IPSC); score range: 18-90

^f^The Self-Efficacy Questionnaire for Phobic Situations (SEQ-SP); score range: 14-70

^g^The Parental Self-Efficacy Questionnaire for Dental Anxiety (P-SEQ-DA); score range:0-120
