# Supplementary material for: Internet-Based Cognitive Behavioral Therapy for Children and Adolescents With Dental or Injection Phobia: Randomized Controlled Trial
Source: J Med Internet Res. 2024 Feb 21;26:e42322. doi: 10.2196/42322 (PMC10918554; doi:10.2196/42322)
Supplement: Multimedia Appendix 6 [file jmir_v26i1e42322_app6.docx]

| Outcome variable | Mean difference | 95% CI | *t* test | | |
| --- | --- | --- | --- | --- | --- |
|  |  |  | *P* value^a^ | Cohen *d* | 95% CI |
| *Child* |  |  |  |  |  |
| Dental procedures managed^b^, n | 3.6 | 2.0 to 5.2 | **<.001** | 1.2 | 0.5 to 1.8 |
| Dental fear and anxiety^c^ | –11.6 | –16.1 to –7.2 | **<.001** | 1.3 | 0.7 to 2.0 |
| Negative cognitions^d^ | –13.3 | –18.7 to –8.0 | **<.001** | 1.4 | 0.7 to 2.1 |
| Injection fear^e^ | –10.6 | –16.0 to –5.3 | **.001** | 1.1 | 0.4 to 1.7 |
| Self-efficacy^f^ | 18.3 | 11.8 to 24.8 | **<.001** | 1.5 | 0.8 to 2.2 |
| *Parent* |  |  |  |  |  |
| Dental procedures managed^b^, n | 3.7 | 1.8 to 5.6 | **.001** | 1.0 | 0.4 to 1.6 |
| Dental fear and anxiety^c^ | –10.9 | –15.7 to –6.1 | **<.001** | 1.2 | 0.5 to 1.8 |
| Parental self-efficacy^g^ | 3.7 | 3.3 to 10.7 | .281 | 0.3 | –0.2 to 0.8 |

^a^P values are based on *t* test

^b^The picture-guided behavioral avoidance test (PG-BAT); score range: 0-17

^c^The Children’s Fear and Survey Schedule – Dental Subscale (CFSS-DS); score range: 15-75

^d^The Children’s Negative Cognitions in Dentistry (CNCD) scale; score range: 0-50

^e^The Injection Phobia Scale for Children (IPSC); score range: 18-90

^f^The Self-Efficacy Questionnaire for Phobic Situations (SEQ-SP); score range: 14-70

^g^The Parental Self-Efficacy Questionnaire for Dental Anxiety (P-SEQ-DA); score range:0-120
