# Supplementary material for: Internet-Based Cognitive Behavioral Therapy for Children and Adolescents With Dental or Injection Phobia: Randomized Controlled Trial
Source: J Med Internet Res. 2024 Feb 21;26:e42322. doi: 10.2196/42322 (PMC10918554; doi:10.2196/42322)
Supplement: Multimedia Appendix 7 [file jmir_v26i1e42322_app7.pdf]

CONSORT-EHEALTH Checklist V1.6.2 Report	Manuscript Number	42322
**(based on CONSORT-EHEALTH V1.6), available at [http://tinyurl.com/consort-ehealth-v1-6].**		
**Date completed**		
2/7/2024 6:15:42		
**by**		
Robert Schibbye		
Internet-Based Cognitive Behavioral Therapy for Children and Adolescents With Dental or Injection Phobia: Randomized Controlled Trial		
TITLE		
**1a-i) Identify the mode of delivery in the title**		
"Internet-Based Cognitive Behavioral Therapy for Children and Adolescents With Dental or Injection Phobia: Randomized Controlled Trial"		
**1a-ii) Non-web-based components or important co-interventions in title**		
**1a-iii) Primary condition or target group in the title**		
"Internet-Based Cognitive Behavioral Therapy for Children and Adolescents With Dental or Injection Phobia: Randomized Controlled Trial"		
ABSTRACT		
**1b-i) Key features/functionalities/components of the intervention and comparator in the METHODS section of the ABSTRACT**		
"ICBT was based on exposure therapy and comprised a 12-week at-home program combined with visits to their regular dental clinic. Participants corresponded weekly with their therapist after completing each module, and one parent was designated as a coach to support the child in the assignments during treatment."		
**1b-ii) Level of human involvement in the METHODS section of the ABSTRACT**		
"Participants corresponded weekly with their therapist after completing each module"		
**1b-iii) Open vs. closed, web-based (self-assessment) vs. face-to-face assessments in the METHODS section of the ABSTRACT**		
"The measurements included a structured diagnostic interview with a clinical psychologist. Our primary outcome measure was the Picture-Guided Behavioral Avoidance Test (PG-BAT), which assesses the ability to approach 17 dental clinical procedures, and a positive clinical diagnosis. Secondary outcome measures included self-report questionnaires that measured self-efficacy and levels of dental and injection anxiety. The children and their parents completed the questionnaires."		
**1b-iv) RESULTS section in abstract must contain use data**		
"After inclusion, participants were randomized to either ICBT (17/33, 52%) or a control group of children on a waitlist (16/33, 48%)", "All participants underwent the 12-week follow-up."		
**1b-v) CONCLUSIONS/DISCUSSION in abstract for negative trials**		
INTRODUCTION		
**2a-i) Problem and the type of system/solution**		
"CBT treatments are widely recognized as the most effective treatment for specific phobias and anxieties, but they are rarely implemented in dental practice [10]. The focus of CBT in the pediatric dental care setting is to reduce patients’ anxiety so that they can willingly receive treatment without the need for sedation or restraint. CBT, which is normally administered face-to-face by a trained psychotherapist, has been shown to be effective for both adults [11,12] and children and adolescents [13-16] with DFA or DP/IP. However, accessibility to and use of CBT are generally low in dentistry. Common barriers include a lack of trained CBT therapists, high costs for the family if the child is treated in private psychotherapeutic services, and time constraints (ie, it might be difficult for parents to take time off from work for an extended period for weekly visits to the psychologist) [17]. In addition, there might be dentistry-specific barriers, such as a lack of knowledge about CBT methods and problems integrating workflows and new personnel (ie, CBT therapists) into the clinic.", "Internet-based CBT (ICBT) was developed to make CBT more accessible."		
**2a-ii) Scientific background, rationale: What is known about the (type of) system**		

"ICBT has been found to be effective for children with specific phobias [20,21] and children and adolescents with DP [22]. These studies have used parents as coaches guiding the child through the treatments.<br>The aim of this study was to test, in a randomized controlled trial (RCT), whether ICBT can reduce fear and increase willingness to receive dental treatment in children and adolescents with DP/IP."		
**Does your paper address CONSORT subitem 2b?**		
"The aim of this study was to test, in a randomized controlled trial (RCT), whether ICBT can reduce fear and increase willingness to receive dental treatment in children and adolescents with DP/IP."		
METHODS		
**3a) CONSORT: Description of trial design (such as parallel, factorial) including allocation ratio**		
"One person uninvolved with the study and blinded to the identities of the participants then randomized the participants consecutively in a 1:1 ratio to active treatment or to a waiting list (the control group) using the list randomization tool at www.random.org and the participants' study ID number. New participants were continuously assigned using a randomized block design; the block size varied depending on the number of participants available at the time of randomization. In case there was 1 participant or an uneven number of participants, a dummy participant was added to the block to maintain a 1:1 ratio for randomization."		
**3b) CONSORT: Important changes to methods after trial commencement (such as eligibility criteria), with reasons**		
"We planned to recruit 50 participants. However, owing to the slower-than-expected recruitment pace, we extended the originally planned recruitment time and ended the recruitment with 33 participants included in the study."		
**3b-i) Bug fixes, Downtimes, Content Changes**		
**4a) CONSORT: Eligibility criteria for participants**		
"Eligible participants who met the inclusion criteria (Textbox 1) were asked to join the study; they all agreed to participate."<br><br>"Participants who met any of the exclusion criteria (Textbox 2) were excluded."		
**4a-i) Computer / Internet literacy**		
Inclusion criteria contained:<br>"• The Swedish language skills of the child and parents are sufficient to manage treatment and the questionnaires.<br>• Access to a computer and the internet is readily available.<br>• Child and parents have sufficient time and motivation to work with internet-based cognitive behavioral therapy 3 hours each week for 12 weeks."		
**4a-ii) Open vs. closed, web-based vs. face-to-face assessments:**		
"Participants were recruited through dental clinics and social media advertisements that referred interested families to a website of the Department of Dental Medicine at Karolinska Institute. The study website contained brief information about the study and the targeted population. Interested parents (or caregivers) applied directly through the website and were then assigned a log-in for web-based screening. We informed dental clinics throughout Sweden and encouraged them to advertise for participants in the waiting rooms. We also asked dentists, especially specialists in pediatric dentistry, to recommend patients with DFA that was so severe that it interfered with dental treatment to apply to the study."<br>"The first step in web-based screening provided information on the study and details about informed consent from the caregivers"<br>"A clinical psychologist then interviewed the parent over the telephone."		
**4a-iii) Information giving during recruitment**		
"Before the participants could complete screening and enter the study, informed consent was obtained. Information about the study and informed consent was provided in Swedish and included permission for secondary analysis of the data without additional consent. Informed consent was provided by both caregivers separately if there were 2. There was no compensation given to the participants, and similar to all dental care for children and adolescents in Sweden, they received the intervention for free."		
**4b) CONSORT: Settings and locations where the data were collected**		
"The intervention and all data collection occurred on a secure web-based platform hosted by the Internetpsykiatri (internet psychiatry) unit run by Stockholm Health Care Services, Region Stockholm, Sweden."		
**4b-i) Report if outcomes were (self-)assessed through online questionnaires**		

"This study had 2 primary outcome measures. The first was the specific phobia section of the K-SADS-PL [25], which we used to diagnose DP and IP among the participants. A clinical psychologist included this in a semistructured interview over the telephone with the parent."<br>"The second primary outcome measure was the PG-BAT [17,22], which the child and parent rated separately."<br>"The children rated the 5-item CNCD scale [22] on a 10-point VAS."<br>"The IPSC [24] was rated by the child and consists of 18 items rated on a 5-point scale ranging from no fear (1) to high fear (5)."<br>"Participants rated the Swedish translation of the 14-item Self-Efficacy Questionnaire for Phobic Situations [26]"<br>"Parents rated a Swedish version of the 12-item Parental Self-Efficacy Questionnaire for Dental Anxiety [22] on a 10-point scale with the following end points: 0=no parental self- efficacy and 10=very high parental self-efficacy."		
**4b-ii) Report how institutional affiliations are displayed**		
"Participants were recruited through dental clinics and social media advertisements that referred interested families to a website of the Department of Dental Medicine at Karolinska Institute."		
**5) CONSORT: Describe the interventions for each group with sufficient details to allow replication, including how and when they were actually administered**		
**5-i) Mention names, credential, affiliations of the developers, sponsors, and owners**		
"The intervention and all data collection occurred on a secure web-based platform hosted by the Internetpsykiatri (internet psychiatry) unit run by Stockholm Health Care Services, Region Stockholm, Sweden."<br><br>There was no ownership, sponsors or commercial interests behind the study.		
**5-ii) Describe the history/development process**		
"The form of ICBT used in this study was based on a previously published manual [17] that was used in a pilot study [22]."		
**5-iii) Revisions and updating**		
No such things. We had one version that we ran with throughout the study. "The form of ICBT used in this study was based on a previously published manual [17] that was used in a pilot study [22]."		
**5-iv) Quality assurance methods**		
**5-v) Ensure replicability by publishing the source code, and/or providing screenshots/screen-capture video, and/or providing flowcharts of the algorithms used**		
 "The form of ICBT used in this study was based on a previously published manual [17] that was used in a pilot study [22]."		
**5-vi) Digital preservation**		
**5-vii) Access**		
"There was no compensation given to the participants, and similar to all dental care for children and adolescents in Sweden, they received the intervention for free. For added security, the secure web-based platform in which data were collected required a 2-step authentication via SMS text message for logging in both for participants and the study staff."		
**5-viii) Mode of delivery, features/functionalities/components of the intervention and comparator, and the theoretical framework**		
"The central component of the treatment was exposure through video and audio recordings, a toolkit for use at home, and in vivo exposure at the dental clinic. Other components of the treatment were psychoeducation, behavioral analyses, controlled breathing, and parental education (Textbox 4)."		
**5-ix) Describe use parameters**		
"Child and parents have sufficient time and motivation to work with internet-based cognitive behavioral therapy 3 hours each week for 12 weeks."		
**5-x) Clarify the level of human involvement**		

"The ICBT intervention comprised 12 web-based treatment modules that were made accessible to the participants, with one module per week. Each participant was assigned a psychologist who supported them throughout the treatment. In total, 3 psychologists were involved in this study; all had a 5-year degree in clinical psychology at a minimum. In addition, all 3 had face-to-face clinical dental experience in CBT with children and adolescents who had DP/IP."<br>"Each week, participants sent their responses to the questions and a log of their assignments to their psychologist, who would send feedback and grant access to the next module within 2 days. Participants could also message their psychologist directly through their account on the platform and expect a reply, on average, within 2 working days. The psychologists would send reminders to inactive participants about continuing work with the modules via SMS text message and email. If participants were inactive for >10 days, the psychologist would try to reach them by telephone."		
**5-xi) Report any prompts/reminders used**		
"The psychologists would send reminders to inactive participants about continuing work with the modules via SMS text message and email. If participants were inactive for >10 days, the psychologist would try to reach them by telephone."		
**5-xii) Describe any co-interventions (incl. training/support)**		
"The central component of the treatment was exposure through video and audio recordings, a toolkit for use at home, and in vivo exposure at the dental clinic. " "During week 2, the participants received a toolkit with dental instruments and a VAS that the children and adolescents and coach could use for the exposure assignments at home (Multimedia Appendix 1)."		
**6a) CONSORT: Completely defined pre-specified primary and secondary outcome measures, including how and when they were assessed**		
The trail was pre-registered. See: https://clinicaltrials.gov/study/NCT02588079		
**6a-i) Online questionnaires: describe if they were validated for online use and apply CHERRIES items to describe how the questionnaires were designed/deployed**		
"The instrument was evaluated in our open trial study on ICBT for dental anxiety in children and adolescents [22]."		
**6a-ii) Describe whether and how “use” (including intensity of use/dosage) was defined/measured/monitored**		
"All participants underwent the 12-week follow-up; thus, no data were missing. Of the 17 participants in the ICBT group, 1 (6%) chose not to continue the intervention after week 2 because of other priorities and lack of time; we categorized this participant as a dropout. The other 94% (16/17) of the participants completed at least 5 modules and started the first steps of exposure training. A total of 81% (13/16) of the participants were considered treatment completers as they finished the most important steps of the treatment (module 8) and were conducting in vivo exposure sessions at their local dental clinic at the time of the follow-up. The mean number of completed modules after 12 weeks was 8.4 (SD 3.4). In total, 12% (2/16) of the participants in the control group chose not to enroll in ICBT after the study was concluded."		
**6a-iii) Describe whether, how, and when qualitative feedback from participants was obtained**		
"Furthermore, the follow-up included a questionnaire concerning qualitative aspects of the child’s current dental anxiety and the ICBT treatment; response options included free text, multiple choice, and visual analog scales (VASs). The questionnaires also contained clear questions about adverse events or unwanted treatment effects, which the psychologists covered in their interviews."		
**6b) CONSORT: Any changes to trial outcomes after the trial commenced, with reasons**		
"The intervention and all data collection occurred on a secure web-based platform hosted by the Internetpsykiatri (internet psychiatry) unit run by Stockholm Health Care Services, Region Stockholm, Sweden."		
**7a) CONSORT: How sample size was determined**		
**7a-i) Describe whether and how expected attrition was taken into account when calculating the sample size**		
"We expected the effects to be in line with our open trial study using the same procedures and treatment [22]. Thus, the power calculation was based on an estimated effect size of Cohen d=1.0 and showed that, to obtain 80% power (Cronbach α=.05), 17 participants in each arm (N=34) were required."		
**7b) CONSORT: When applicable, explanation of any interim analyses and stopping guidelines**		
The trail was pre-registered. See: https://clinicaltrials.gov/study/NCT02588079		
**8a) CONSORT: Method used to generate the random allocation sequence**		
We only had one active trail group so no allocation of care providers between groups was needed.		
**8b) CONSORT: Type of randomisation; details of any restriction (such as blocking and block size)**		

"One person uninvolved with the study and blinded to the identities of the participants then randomized the participants consecutively in a 1:1 ratio to active treatment or to a waiting list (the control group) using the list randomization tool at www.random.org and the participants' study ID number. New participants were continuously assigned using a randomized block design; the block size varied depending on the number of participants available at the time of randomization. In case there was 1 participant or an uneven number of participants, a dummy participant was added to the block to maintain a 1:1 ratio for randomization."		
**9) CONSORT: Mechanism used to implement the random allocation sequence (such as sequentially numbered containers), describing any steps taken to conceal the sequence until interventions were assigned**		
"One person uninvolved with the study and blinded to the identities of the participants then randomized the participants consecutively in a 1:1 ratio to active treatment or to a waiting list (the control group) using the list randomization tool at www.random.org and the participants' study ID number. New participants were continuously assigned using a randomized block design; the block size varied depending on the number of participants available at the time of randomization. In case there was 1 participant or an uneven number of participants, a dummy participant was added to the block to maintain a 1:1 ratio for randomization."		
**10) CONSORT: Who generated the random allocation sequence, who enrolled participants, and who assigned participants to interventions**		
"One person uninvolved with the study and blinded to the identities of the participants then randomized the participants consecutively in a 1:1 ratio to active treatment or to a waiting list (the control group) using the list randomization tool at www.random.org and the participants' study ID number."		
**11a) CONSORT: Blinding - If done, who was blinded after assignment to interventions (for example, participants, care providers, those assessing outcomes) and how**		
**11a-i) Specify who was blinded, and who wasn't**		
The clinician assessing the participants post-treatment was not the one who had been in contact with the participant throughout the study. However the plattform itself was not blinded to the clinicians, making it a possibility that they might have seen the names of the participants in the platform before calling them. We never assessed the blindness of the clinician after the interview which we discuss as a weakness of the study in the article:<br>"The blinding of the clinicians who performed the follow-up interview was not assessed in this study."		
**11a-ii) Discuss e.g., whether participants knew which intervention was the "intervention of interest" and which one was the "comparator"**		
Ww used a waiting list design, so not applicable.		
**11b) CONSORT: If relevant, description of the similarity of interventions**		
Ww used a waiting list design, so not applicable.		
**12a) CONSORT: Statistical methods used to compare groups for primary and secondary outcomes**		
"All statistical analyses were conducted using SPSS (version 27; IBM Corp). We accepted a 5% type-I error in all analyses. To compare the means between the 2 study conditions, we used t tests. Repeated-measure ANOVAs were conducted to evaluate possible differences in changes over the 12 weeks (baseline to follow-up) between the ICBT and control groups. We estimated the effect size using the Cohen d, that is, the standardized mean difference [27]. Chi-square tests of independence were conducted to evaluate possible between-group differences in meeting the diagnostic criteria. Before conducting our analyses, we checked the data for normality."		
**12a-i) Imputation techniques to deal with attrition / missing values**		
"All participants underwent the 12-week follow-up; thus, no data were missing."		
**12b) CONSORT: Methods for additional analyses, such as subgroup analyses and adjusted analyses**		
Not applicable, no subgroup analyses were made.		
RESULTS		
**13a) CONSORT: For each group, the numbers of participants who were randomly assigned, received intended treatment, and were analysed for the primary outcome**		
Yes, see the CONSORT Flow Diagram in the article.		
**13b) CONSORT: For each group, losses and exclusions after randomisation, together with reasons**		
Yes, see the CONSORT Flow Diagram in the article.		
**13b-i) Attrition diagram**		

"All participants underwent the 12-week follow-up; thus, no data were missing. Of the 17 participants in the ICBT group, 1 (6%) chose not to continue the intervention after week 2 because of other priorities and lack of time; we categorized this participant as a dropout. The other 94% (16/17) of the participants completed at least 5 modules and started the first steps of exposure training. A total of 81% (13/16) of the participants were considered treatment completers as they finished the most important steps of the treatment (module 8) and were conducting in vivo exposure sessions at their local dental clinic at the time of the follow-up. The mean number of completed modules after 12 weeks was 8.4 (SD 3.4). In total, 12% (2/16) of the participants in the control group chose not to enroll in ICBT after the study was concluded."		
**14a) CONSORT: Dates defining the periods of recruitment and follow-up**		
"Participants were recruited from October 2015 to December 2019"		
**14a-i) Indicate if critical "secular events" fell into the study period**		
None happened, so not applicable.		
**14b) CONSORT: Why the trial ended or was stopped (early)**		
It did not stop early. We prolonged the recruitment and stopped it once the minimum number of persons included according to our power calculations.		
**15) CONSORT: A table showing baseline demographic and clinical characteristics for each group**		
"Table 1 presents the participant characteristics."<br>"In total, 3 psychologists were involved in this study; all had a 5-year degree in clinical psychology at a minimum. In addition, all 3 had face-to-face clinical dental experience in CBT with children and adolescents who had DP/IP."		
**15-i) Report demographics associated with digital divide issues**		
Since we had inclusion criteria about participants having internet and sufficient time and motivation to work with the treatment 3 hours each week for 12 weeks some people might have been excluded. We discuss this in the article<br>"Speculatively, some families may have too few resources or be reluctant to commit to a program as long as 12 weeks. This is further exemplified by one participant in the study who dropped out because of time constraints and 3 participants who did not complete the modules during the treatment time. The extra burden of investing time in a treatment of this type makes it unsuitable for some families and individuals. This limits the use of both CBT and ICBT in its current form, and more research is needed on how to provide a feasible, evidence-based alternative for this group. Perhaps one alternative that should be tested is an even shorter course in exposure- based CBT that is less text dependent."		
**16a) CONSORT: For each group, number of participants (denominator) included in each analysis and whether the analysis was by original assigned groups**		
**16-i) Report multiple "denominators" and provide definitions**		
Yes, see the CONSORT Flow Diagram in the article.<br><br>"All participants underwent the 12-week follow-up; thus, no data were missing. Of the 17 participants in the ICBT group, 1 (6%) chose not to continue the intervention after week 2 because of other priorities and lack of time; we categorized this participant as a dropout. The other 94% (16/17) of the participants completed at least 5 modules and started the first steps of exposure training. A total of 81% (13/16) of the participants were considered treatment completers as they finished the most important steps of the treatment (module 8) and were conducting in vivo exposure sessions at their local dental clinic at the time of the follow-up. The mean number of completed modules after 12 weeks was 8.4 (SD 3.4). In total, 12% (2/16) of the participants in the control group chose not to enroll in ICBT after the study was concluded."		
**16-ii) Primary analysis should be intent-to-treat**		
"We used an intention-to-treat design; that is, participants were included in the analyses irrespective of the extent to which they had completed the treatment."		
**17a) CONSORT: For each primary and secondary outcome, results for each group, and the estimated effect size and its precision (such as 95% confidence interval)**		
Yes, see tables in results section.		
**17a-i) Presentation of process outcomes such as metrics of use and intensity of use**		

"All participants underwent the 12-week follow-up; thus, no data were missing. Of the 17 participants in the ICBT group, 1 (6%) chose not to continue the intervention after week 2 because of other priorities and lack of time; we categorized this participant as a dropout. The other 94% (16/17) of the participants completed at least 5 modules and started the first steps of exposure training. A total of 81% (13/16) of the participants were considered treatment completers as they finished the most important steps of the treatment (module 8) and were conducting in vivo exposure sessions at their local dental clinic at the time of the follow-up. The mean number of completed modules after 12 weeks was 8.4 (SD 3.4). In total, 12% (2/16) of the participants in the control group chose not to enroll in ICBT after the study was concluded."		
**17b) CONSORT: For binary outcomes, presentation of both absolute and relative effect sizes is recommended**		
"In the ICBT group, 41% (7/17) of the participants no longer met the diagnostic criteria for DP, IP, or DP/IP at the posttreatment clinical interview compared with 0% in the control group. A chi-square test of independence showed that the difference was significant (χ21=8.4, P=.004). A total of 65% (11/17) of the participants lost at least one of their earlier diagnoses of either DP or IP (N=33, χ21=15.5, P<.001) compared with 0% in the control group. Table 3 presents the results of the intervention stratified by diagnosis."		
**18) CONSORT: Results of any other analyses performed, including subgroup analyses and adjusted analyses, distinguishing pre-specified from exploratory**		
No other analysis were made.		
**18-i) Subgroup analysis of comparing only users**		
"Multimedia Appendix 5 presents a complete table of means and SDs at follow-up for all outcome measurements stratified by allocation group.<br>"Multimedia Appendix 6 presents within-group effects for the ICBT group; no within-group effects were observed in the control group."		
**19) CONSORT: All important harms or unintended effects in each group**		
"Participants reported no adverse events or unintended effects during the intervention."		
**19-i) Include privacy breaches, technical problems**		
Some participants forgot their log-information for follow-ups or at intervention start after being on the waiting list, but it was sent out automatically through sms, to minor to mention in article.		
**19-ii) Include qualitative feedback from participants or observations from staff/researchers**		
Because of constraints from article length and scope, no qualitative feedback is reported in this article.		
DISCUSSION		
**20) CONSORT: Trial limitations, addressing sources of potential bias, imprecision, multiplicity of analyses**		
**20-i) Typical limitations in ehealth trials**		
"Regarding limitations, the control group in this study was a waiting list. We initially hoped to establish the efficacy of ICBT treatment before testing it against active control conditions or establish noninferiority by comparing it with face-to-face CBT. The blinding of the clinicians who performed the follow-up interview was not assessed in this study. In addition, the study had a fairly small sample size, which precludes, for example, meaningful subgroup analyses."<br><br>"Another limitation of this study is that recruitment through advertisements might not reach the general population, creating a nonrepresentative group. This could be another factor behind the slow recruitment, suggesting treatment barriers for certain groups that warrant further investigation. Speculatively, some families may have too few resources or be reluctant to commit to a program as long as 12 weeks. This is further exemplified by one participant in the study who dropped out because of time constraints and 3 participants who did not complete the modules during the treatment time. The extra burden of investing time in a treatment of this type makes it unsuitable for some families and individuals. This limits the use of both CBT and ICBT in its current form, and more research is needed on how to provide a feasible, evidence-based alternative for this group. Perhaps one alternative that should be tested is an even shorter course in exposure-based CBT that is less text dependent."		
**21) CONSORT: Generalisability (external validity, applicability) of the trial findings**		
**21-i) Generalizability to other populations**		

"This study also had high external validity as the dental treatment and in vivo exposure were administered at general dental clinics by personnel with no formal training in CBT. Finally, the sample was clinically relevant, having had fears of visiting the dentist for an extended time and previous experience of unsuccessful treatment to reduce their DFA or DP/IP through sedation, restraint, or general anesthesia."<br><br>"Another limitation of this study is that recruitment through advertisements might not reach the general population, creating a nonrepresentative group. This could be another factor behind the slow recruitment, suggesting treatment barriers for certain groups that warrant further investigation. Speculatively, some families may have too few resources or be reluctant to commit to a program as long as 12 weeks. This is further exemplified by one participant in the study who dropped out because of time constraints and 3 participants who did not complete the modules during the treatment time. The extra burden of investing time in a treatment of this type makes it unsuitable for some families and individuals. This limits the use of both CBT and ICBT in its current form, and more research is needed on how to provide a feasible, evidence-based alternative for this group. Perhaps one alternative that should be tested is an even shorter course in exposure-based CBT that is less text dependent."<br><br>"Finally, this study was conducted in Sweden, which has publicly funded, free-of-charge dental services for children. The generalizability of the findings to other dental health care contexts needs to be further investigated. More specifically, how the cost of this type of treatment might influence its acceptability for the patient and dentist in different contexts needs to be explored."		
**21-ii) Discuss if there were elements in the RCT that would be different in a routine application setting**		
"This study also had high external validity as the dental treatment and in vivo exposure were administered at general dental clinics by personnel with no formal training in CBT. Finally, the sample was clinically relevant, having had fears of visiting the dentist for an extended time and previous experience of unsuccessful treatment to reduce their DFA or DP/IP through sedation, restraint, or general anesthesia."		
**22) CONSORT: Interpretation consistent with results, balancing benefits and harms, and considering other relevant evidence**		
**22-i) Restate study questions and summarize the answers suggested by the data, starting with primary outcomes and process outcomes (use)**		
"After treatment, 41% (7/17) of the participants in the ICBT group no longer met the diagnostic criteria for either DP or IP, whereas all participants in the control group did. Furthermore, an additional 24% (4/17) of children in the ICBT group no longer met the diagnostic criteria for one of their 2 diagnoses at baseline, totaling 65% (11/17) of children in the ICBT group in whom at least one diagnosis had remitted. Compared with the control group, participants in the ICBT group could also willingly receive significantly more treatment steps at the dentist, and their fear, anxiety, and negative cognitions toward dentistry and injections significantly decreased after 12 weeks. As rated by both the children and parents, the effect size calculated from the primary outcome (PG-BAT) was large. In addition, children in the ICBT group reported higher self-efficacy. In summary, this RCT indicates that ICBT is an effective treatment for children with DP and IP."		
**22-ii) Highlight unanswered new questions, suggest future research**		
"Taken together, there is an emerging base of evidence for exposure-based CBT in pediatric dentistry, and it seems that this type of treatment can be delivered effectively in several ways. An important avenue for future research is to investigate whether ICBT also produces effects superior to those of active control conditions and noninferiority compared with traditional face-to-face CBT."<br>"Finally, this study excluded participants with neurodevelopmental disorders, a group of patients that is common in specialist pediatric dentistry. Future research needs to explore how exposure-based treatments can be successfully adapted for these patients."<br>"The extra burden of investing time in a treatment of this type makes it unsuitable for some families and individuals. This limits the use of both CBT and ICBT in its current form, and more research is needed on how to provide a feasible, evidence-based alternative for this group. Perhaps one alternative that should be tested is an even shorter course in exposure- based CBT that is less text dependent."<br>"The generalizability of the findings to other dental health care contexts needs to be further investigated. More specifically, how the cost of this type of treatment might influence its acceptability for the patient and dentist in different contexts needs to be explored."		
Other information		
**23) CONSORT: Registration number and name of trial registry**		
"https://clinicaltrials.gov/study/NCT02588079", "NCT02588079"		
**24) CONSORT: Where the full trial protocol can be accessed, if available**		
No other then the information in the article and the pre-registration of the trail available at: https://clinicaltrials.gov/study/NCT02588079 and the submitted documents in the ethical approval from the regional ethics review board of Stockholm (ID 2014/633-31/5).		
**25) CONSORT: Sources of funding and other support (such as supply of drugs), role of funders**		
No external funding sources or sponsors.		

**X26-i) Comment on ethics committee approval**		
"Ethical Considerations<br>Before the participants could complete screening and enter the study, informed consent was obtained. Information about the study and informed consent was provided in Swedish and included permission for secondary analysis of the data without additional consent. Informed consent was provided by both caregivers separately if there were 2. There was no compensation given to the participants, and similar to all dental care for children and adolescents in Sweden, they received the intervention for free. For added security, the secure web-based platform in which data were collected required a 2-step authentication via SMS text message for logging in both for participants and the study staff. When extracting data from the platform for analysis, they were deidentified, and an anonymous study ID was used as an identifier for each participant. The regional ethics review board of Stockholm approved this study (ID 2014/633-31/5)."		
**x26-ii) Outline informed consent procedures**		
"The first step in web-based screening provided information on the study and details about informed consent from the caregivers"		
**X26-iii) Safety and security procedures**		
"Participants could also message their psychologist directly through their account on the platform and expect a reply, on average, within 2 working days." "For added security, the secure web-based platform in which data were collected required a 2-step authentication via SMS text message for logging in both for participants and the study staff. When extracting data from the platform for analysis, they were deidentified, and an anonymous study ID was used as an identifier for each participant."		
**X27-i) State the relation of the study team towards the system being evaluated**		
Same research team that developed the treatment that conducted the study. But no external funding sources or sponsors or commercial interests in the treatment.		
